# Towards theoretical foundation for high-precision fringe projection profilometry system design and optimization

**DOI:** 10.1038/s41377-026-02393-4

**Published:** 2026-06-26

**Authors:** Song Zhang

**Affiliations:** School of Mechanical Engineering, Purdue University, West Lafayette, IN USA

**Keywords:** Imaging and sensing, Optical sensors

## Abstract

A rigorous theoretical foundation for the design, evaluation, and optimization of high-precision FPP systems is critically needed. A recent article published in *Light:* *Science & Applications* marks an important step toward this goal.

Fringe projection profilometry (FPP) is a triangulation-based three-dimensional (3D) optical metrology technique widely used for accurate 3D surface measurement. Compared with other optical 3D measurement techniques, FPP offers several unique advantages, including high temporal and spatial resolutions, high measurement accuracy, strong flexibility, and relative robustness to surface texture variations^1,2^. Over the past decades, extensive research efforts have focused on improving the speed, flexibility, and accessibility of FPP systems. As a result, FPP has been widely adopted in applications such as manufacturing, healthcare, robotics and automation, cultural heritage preservation, and digital archiving, among many others^1,2^.

Despite these advancements, developing a high-performance FPP system remains challenging because reconstruction quality depends critically on both hardware and software design^3^. The problem is further complicated by the numerous factors that influence reconstruction precision, including sensor noise, phase quality, system geometry, optical distortion, and lens characteristics. As a result, FPP system design and optimization remain largely empirical and heavily reliant on expert experience, lacking a rigorous and unified theoretical framework. Establishing such a foundation for precision-aware system design remains insufficiently explored, yet is essential for the continued advancement of the field.

Lv and Kemao^4^ developed a comprehensive theoretical framework for FPP using vertical fringes (Ver3), establishing a predictive model that quantitatively characterizes noise propagation from camera acquisition to final 3D reconstruction. By introducing a non-Gaussian camera noise model, they directly connected camera parameters with phase and geometric precision prediction. They further derived explicit phase-to-geometry transfer models and simplified analytical formulations that reveal how key system parameters, including baseline, focal length, fringe period, and measurement distance, influence reconstruction precision.

Building upon this prior work, Lv et al.^5^ recently developed a unified theoretical framework for understanding and evaluating the precision of FPP systems (Fig. 1), published in *Light: Science & Applications*. In this study, they derived a general precision model that unifies the three major FPP reconstruction approaches—Ver3, horizontal fringes (Hor3), and optimal-angle fringes (OptE3)—within a single mathematical formulation. This unification revealed an elegant Pythagorean relationship among the precisions of these methods and further demonstrated that the OptE3 approach achieves the highest precision under identical system configurations. While previous studies experimentally demonstrated the advantages of optimal fringe angles^6^, this work provides one of the first rigorous theoretical explanations for why such angles maximize reconstruction precision and how system parameters can be optimized accordingly. The study further simplified the general model into more physically intuitive forms, revealing the critical roles of effective baseline and optical-axis angle in determining measurement precision. These simplified formulations provide intuitive geometric insight and practical guidance for precision-aware system design and optimization.

**Fig. 1:**
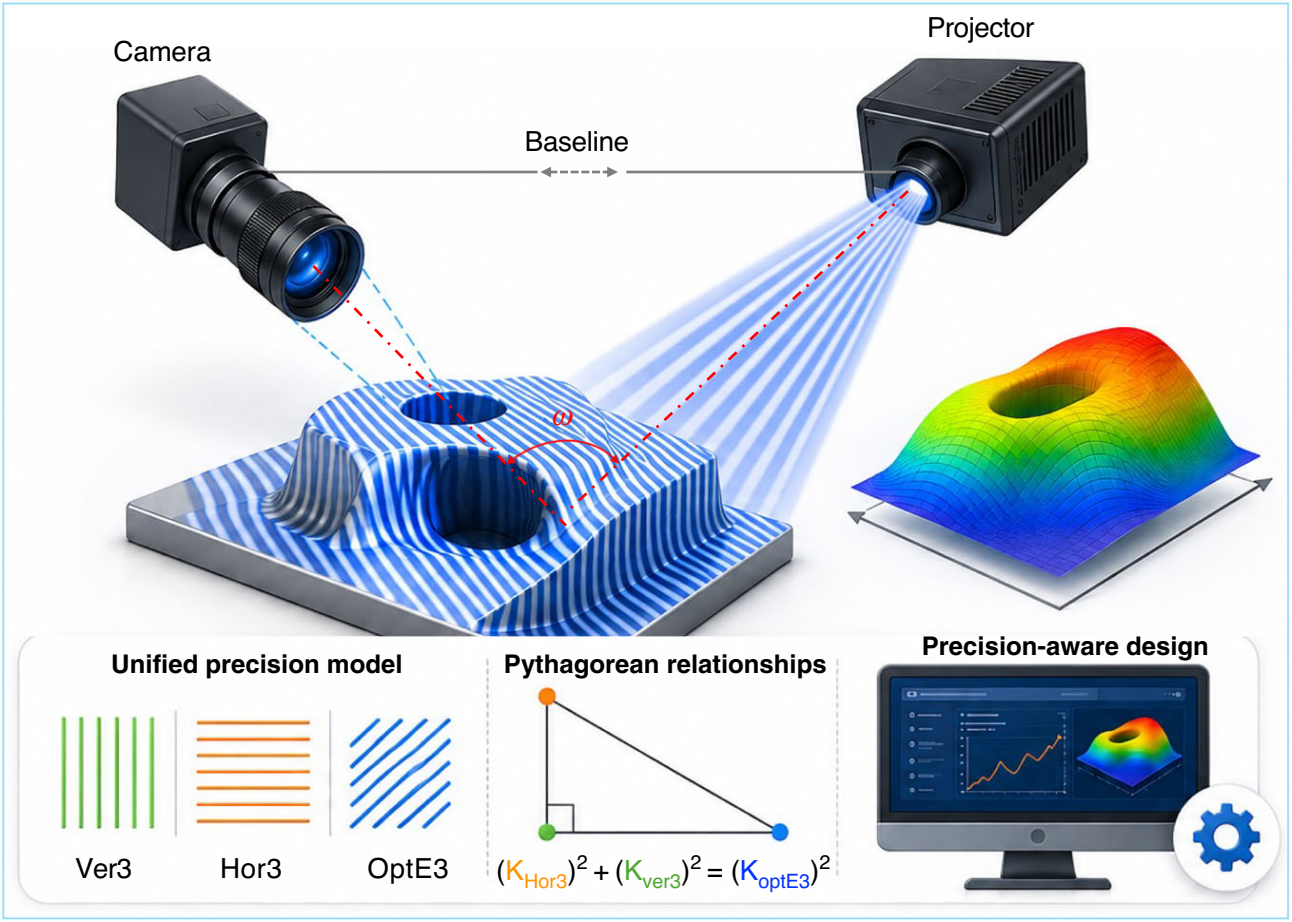
The unified theoretical models and the Planner software tool for FPP system design, evaluation, and optimization

Beyond theoretical unification, the authors also developed an FPP-Planner software tool for precision-aware system design and optimization. This tool estimates the system parameters required to satisfy specified precision targets, thereby bridging the gap between theoretical analysis and practical engineering implementation. In addition, the authors demonstrated other triangulation-based techniques (i.e., stereo vision, laser triangulation) share equivalent precision characteristics under appropriate conditions. This generalization significantly broadens the impact of the work by providing theoretical guidance not only for FPP systems, but also for a wider class of triangulation-based 3D imaging technologies. Experimental results were presented to validate the proposed framework, although broader experimental validation under more diverse conditions would further strengthen the generality of the framework.

While this work establishes an important theoretical foundation for FPP, the current framework primarily focuses on ideal pinhole-lens systems without accounting for lens distortion and is limited to single-camera, single-projector configurations. Several important challenges therefore remain, including the incorporation of nonlinear lens distortion, extension to telecentric optical systems, and adaptation to multi-projector and multi-camera configurations. In addition, practical factors such as thermal effects and motion artifacts may significantly influence real-world system performance, highlighting the need for a more comprehensive theoretical framework that extends beyond idealized conditions.

Beyond extending the current theoretical framework, emerging computational technologies may further accelerate the evolution of FPP system designs. Rapid advances in artificial intelligence (AI)^7^, edge AI^8^, computational imaging^9^, and differentiable imaging^10^ are expected to drive new developments in FPP technologies. These emerging directions may enable AI-assisted system optimization, uncertainty quantification, multimodal sensing integration, and intelligent comprehensive optimization frameworks, among many other possibilities.

These advances could ultimately transform FPP system design from a largely empirical engineering process into a theoretically optimized and intelligent sensing design framework.
